# Coupling and Coordination Degrees of the Core Water–Energy–Food Nexus in China

**DOI:** 10.3390/ijerph16091648

**Published:** 2019-05-11

**Authors:** Shasha Xu, Weijun He, Juqin Shen, Dagmawi Mulugeta Degefu, Liang Yuan, Yang Kong

**Affiliations:** 1 Business School, Hohai University, Nanjing 211100, China; xushasha@hhu.edu.cn (S.X.); jqshen@hhu.edu.cn (J.S.); 2 College of Economic & Management, Three Gorges University, Yichang 443002, China; dagmawi.degefu@ryerson.ca (D.M.D.); 2016110710181@ctgu.edu.cn (Y.K.); 3 Faculty of Engineering and Architectural Science, Ryerson University, Toronto, ON M5B 2K3, Canada

**Keywords:** core water–energy–food nexus, coupling degree, coordination degree

## Abstract

Achieving sustainable development in the water–energy–food (WEF) nexus is gaining global attention. The coupling and coordination degrees are a way to measure sustainable development levels of a complex system. This study assessed the coupling and coordination degrees of the core WEF nexus and identified key factors that affect sustainable development. First, an index system for assessing coupling and coordination degrees of the core WEF nexus was built. Second, the development levels of three subsystems as well as the coupling and coordination degrees of the core WEF nexus in China were calculated. The results showed that from 2007 to 2016, the mean value of the coupling degree was 0.746 (range (0.01, 1)), which was a high level. This proved that the three resources were interdependent. Hence, it was necessary to study their relationship. However, the mean value of the coordination degree was 0.395 (range (0, 1)), which was a low level. This showed that the coordination development of the core WEF nexus in China was low. It is necessary to take some measures to improve the situation. According to the key factors that affect the development levels of water, energy, and food subsystems, the authors put forward some suggestions to improve the coordination development of the WEF system in China.

## 1. Introduction

Issues associated with the supply and demand for water, energy, and food have attracted global attention. In January 2011, the Global Risk Report stated that the water–energy–food (WEF) nexus is one of the three important areas that need to be given priority [1]. In November of the same year, the water–energy–food nexus and security were discussed at the United Nations Climate Conference held in Bonn, Germany [2]. The interactions between WEF sectors are undoubtedly attracting the interest of researchers from multidisciplinary areas. This interest is expected to increase in the upcoming decades with increasing population numbers, water shortages, and energy demand [3].

**Figure 1:**
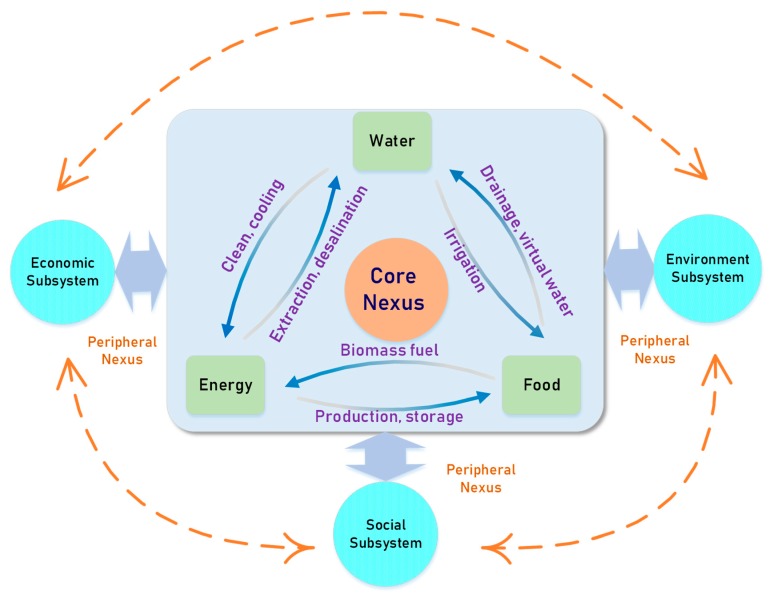
Water–energy–food (WEF) nexus. This figure includes the core and peripheral nexus of the WEF system. The core nexus refers to the relationship among the water, energy, and food subsystems. The peripheral nexus refers to the relationship between the core nexus and social, economic, and environment subsystems.

The WEF nexus is divided into core nexus and peripheral nexus. The core nexus refers to the relationships between the three resources. It is the essence of the WEF nexus. Water, energy, and food are inextricably interrelated and depend on each other [2], which is shown in Figure 1. The withdrawal, treatment, and distribution of water are inseparable from energy. The production of energy also needs water for cleaning and cooling. In the same way, food provides biomass for energy production, and the energy is needed for the production, transportation, and storage of food. Irrigation is an important way to increase food yield. Food growing also helps purify sewage and improve groundwater quality. For some regions, importing food is virtually equivalent to importing the water that would be needed for producing the food locally [4]. Peripheral nexus refers to the correlation of the WEF system with other systems like the ecological, economic, and social systems [5].

There is an extremely fragile, dynamic balance in the WEF system. Any intervention or management measure that has not undergone detailed analysis and scientific assessment is likely to affect or even destroy the system’s balance and cause serious consequences [5]. Synergetic development of the three resources is very important for the achievement of the sustainable goal. The development of nexus synergies to balance food, energy, and water resource trade-offs has been demonstrated by nexus simulation studies [6,7]. Hence, it is of great significance to evaluate the coupling and coordination degrees of the core WEF nexus and identify the causes of system imbalance in order to improve sustainable development.

Most related studies focus on conceptual interpretation of the WEF nexus and the description of its current situation. There is much research on quantitative evaluation of the WEF nexus. A considerable number of studies have quantitatively studied the pairwise relationship between water, energy, and food [8,9,10,11,12,13,14,15,16,17,18,19,20,21,22,23,24,25]. Other studies quantitatively analyzed the WEF nexus with system dynamics [5], input–output approaches [26], water footprints [27], and so on. Beyond that, research on the WEF nexus usually focuses on the relationship among the WEF system and other systems [28,29,30]. However, the core WEF nexus has not gotten enough attention yet [26,31,32,33]. Only with a clear understanding of the essential connection between the three resources can the nexus approach be better applied to broader areas, such as water allocation, life cycle assessment, carbon footprint, water footprint, and supply chain management [34].

Thus, it is important to study the coupling and coordination degrees of the three resources as a system to understand their synergies and ensure sustainable development. Coupling refers to the phenomenon when two or more systems interact with each other. Coupling degree is a measure of the degree of interaction [35]. Coordination is a state or result of system evolution [36]. Since 1999, coupling and coordination degree models have been applied in various research areas [37,38,39,40,41]. It has developed into a mature model suitable for evaluating the coupling and coordination relationships of complex systems.

With population growth, environmental degradation, resource shortages, and intensified impacts of climate change, China as a developing and populous country is facing synergetic issues with water, energy, and food development. The country’s water, energy, and food production systems are unevenly distributed. This is one of the obstacles in achieving sustainable development of economic and social sectors. Guijun et al. (2016) built the system dynamics model of the WEF system for its sustainable development of Beijing [42]. Shaoming et al. (2017) provided the overall analysis framework of the WEF system for collaborative optimization in the Yellow River watershed [43]. However, to the best of our knowledge, the study of coupling and coordination degrees of the core WEF nexus is rare, especially at the national level. Bearing this in mind, this study modeled the coupling and coordination degrees of the core WEF nexus to assess sustainable development of the three resource systems at the national level. This study sought to increase the understanding of the core WEF nexus by calculating its coupling and coordination degrees, and it proposed measures to improve the sustainability of the WEF system.

## 2. Methods

### 2.1. Evaluation Index System for Measuring Coupling and Coordination Degrees of the Core Water–Energy–Food (WEF) Nexus

It is necessary to comprehensively analyze and evaluate multiple indexes to measure the coupling and coordination degrees of the core WEF nexus. Based on existing relevant research on the core WEF nexus [41,44,45], an evaluation index system for measuring the coupling and coordination degrees of the core WEF nexus was constructed (see Table 1).

In this study, positive attribute indexes referred to those indicators that promoted sustainable development of the WEF system. Negative attribute indexes referred to those indicators that could not promote sustainable development of the WEF system. For example, the more industrial water consumption there was, the less energy and food water consumption there was. Industrial water consumption could not promote sustainable development of the WEF system, so it was negative.

### 2.2. Coupling and Coordination Degrees of the Core WEF Nexus

Methodology in this section can be divided into two parts: (1) weighting each index with the entropy value method, and (2) modeling the coupling and coordination degrees of the core WEF nexus. The coupling degree in this paper referred to the extent of various interactions among the three subsystems. The coordination degree emphasized the state and result of subsystem interactions: the higher the coordination degree of three subsystems, the higher the efficiency of the WEF systems.

#### 2.2.1. Weighting Index

Determination of index weight was crucial. A subjective weighting method could not distinctly separate the indices because of its high uncertainty [38]. Therefore, the authors chose the entropy value method as the weighting method.

Implementation of the entropy value method in this study adopted the following procedure:

1. Index selection—suppose there were I subsystems, R years, N regions, and M indicators, then $x_{\mathrm{irnm}}$ was the mth indicator of nth region of the ith subsystem in the rth year.
2. Data standardization—because the indicators had different dimensions and units, they needed to be standardized. At the same time, in order to avoid meaningless logarithms in entropy calculations, non-zero processing was carried out on the data.
  If $x_{\mathrm{irnm}}$ is a positive indicator:
  (1) $$x_{\mathrm{irnm}}^{\prime }=\frac{x_{\mathrm{irnm}}-\min\left(x_{\mathrm{im}}\right)}{\max\left(x_{\mathrm{im}}\right)-\min\left(x_{\mathrm{im}}\right)}\times 0.99+0.01.$$
  If $x_{\mathrm{irnm}}$ is a negative indicator:
  (2) $$x_{\mathrm{irnm}}^{\prime }=\frac{\max\left(x_{\mathrm{im}}\right)-x_{\mathrm{irnm}}}{\max\left(x_{\mathrm{im}}\right)-\min\left(x_{\mathrm{im}}\right)}\times 0.99+0.01,$$
  where $\min\left(x_{\mathrm{im}}\right)\mathrm{and}\;\max\left(x_{\mathrm{im}}\right)$ are the minimum and maximum of the mth index of the ith subsystem in all regions for the selected years, respectively; and $x_{\mathrm{irnm}}^{\prime }$ is a standardized value, with a range of [0.01, 1].
3. The proportion $y_{\mathrm{irnm}}$ of the mth index is indicated by:
  (3) $$y_{\mathrm{irnm}}=\frac{x_{\mathrm{irnm}}^{\prime }}{\sum_{r=1}^{R}\sum_{n=1}^{N}x_{\mathrm{irnm}}^{\prime }}.$$
4. The entropy value $e_{\mathrm{im}}$ of the mth index is indicated by:
  (4) $$e_{\mathrm{im}}=-k\sum_{r=1}^{R}\sum_{n=1}^{N}y_{\mathrm{irnm}}\ln\left(y_{\mathrm{irnm}}\right),$$
  where $k>0\;\mathrm{and}\;k=\frac{1}{\ln\left(\mathrm{RN}\right)}$.
5. The information utility value $g_{\mathrm{im}}$ of the index m is given by:
  (5) $$g_{\mathrm{im}}=1-e_{\mathrm{im}}.$$
6. The weight $w_{\mathrm{im}}$ of the mth index is given by:
  (6) $$w_{\mathrm{im}}=\frac{g_{\mathrm{im}}}{\sum_{m=1}^{M}g_{\mathrm{im}}}.$$

#### 2.2.2. Coupling Degree of the Core WEF Nexus

The evaluation value $f_{\mathrm{irnm}}$ of the mth indicator of the nth region of the ith subsystem in the rth year is indicated by:

(7) $$f_{\mathrm{irnm}}=w_{\mathrm{im}}\times x_{\mathrm{irnm}}^{\prime }.$$

The development level $f_{\mathrm{irn}}$ of the nth region of the ith subsystem in the rth year is indicated by:

(8) $$f_{\mathrm{irn}}=\sum_{m=1}^{M}f_{\mathrm{irnm}}.$$

The contribution $Q_{\mathrm{im}}$ of the mth indicator to the development level of the ith subsystem is given as:

(9) $$Q_{\mathrm{im}}=\frac{f_{\mathrm{im}}}{\sum_{r=1}^{R}\sum_{n=1}^{N}f_{\mathrm{irnm}}}.$$

The mathematical formulations for the coupling degree of the core WEF nexus is shown as follows [15]:

(10) $$C_{\mathrm{rn}}={\left\{\frac{3\left(f_{1\mathrm{rn}}\times f_{2\mathrm{rn}}+f_{1\mathrm{rn}}\times f_{3\mathrm{rn}}+f_{2\mathrm{rn}}\times f_{3\mathrm{rn}}\right)}{{\left(f_{1\mathrm{rn}}+f_{2\mathrm{rn}}+f_{3\mathrm{rn}}\right)}^{2}}\right\}}^{3},$$

where $f_{1\mathrm{rn}}$, $f_{2\mathrm{rn}}$, and $f_{3\mathrm{rn}}$ are the development levels of water, energy, and food subsystems, respectively, in the nth region and the rth year, and their values reflect the relative development levels of the three subsystems. $C_{rn}$ is the coupling degree of the core WEF nexus in the nth region and the rth year, and $C_{rn}\in \left[0,\;1\right]$. The coupling degree $C_{rn}$ is divided into four types (see Table 2) [39].

#### 2.2.3. Coordination Degree of the Core WEF Nexus

The coordination degree was used to reflect the interaction result, which referred to the synchronized development level of the subsystems. While the coupling degree reflectes the interaction process, it is difficult to use the coupling degree to reflect the actual sustainable development level of the core WEF nexus. Therefore, judging the sustainable development level of the core WEF nexus with only the coupling degree function C would likely lead to results that were inconsistent with reality. In order to objectively reflect the interactions of the three subsystems, it was necessary to build a coordination degree model. Therefore, based on the coupling degree function, the mathematical formulations for the coordination degree are shown as follows [39].

(11) $$D_{\mathrm{rn}}=\sqrt{C_{\mathrm{rn}}T_{\mathrm{rn}}},$$

(12) $$T_{\mathrm{rn}}={\mathrm{af}}_{1\mathrm{rn}}+{\mathrm{bf}}_{2\mathrm{rn}}+{\mathrm{cf}}_{3\mathrm{rn}},$$

where $D_{\mathrm{rn}}$ is the coordination degree of the core WEF nexus in the nth region and the rth year, and its range is (0, 1); $C_{\mathrm{rn}}$ is the coupling degree; $T_{\mathrm{rn}}$ is the integrated value of the core WEF nexus, which reflects the integrated assessment value of each region; and a, b, and c represent the contribution of each subsystem to the development level of the system, respectively, and can be obtained by using the entropy value method.

According to the studies [5,46,47], coordination degree can be divided into four types (see Table 3) [39].

### 2.3. Study Area and Data

China is a developing country with a large population. Annual availability of water per person is 2100 m^3^, which is nearly one-quarter of the global average. The distribution of water resources in China is highly asymmetric. There is also severe inconsistency in the spatial distributions of water availability and water demand in the country. This country’s water ecology and environment are fragile.

China has been the largest energy consumer for the last 17 consecutive years in the world. China’s primary energy consumption reached 3132.2 million tons of oil, accounting for 23% of the world’s total energy consumption. The country’s oil production was 191.5 million tons of oil, while consumption was 608.4 million tons of oil. Natural gas production was 149.2 billion m^3^, while consumption was 240.4 billion m^3^. The output of coal was equal to 1747.2 million tons of oil, while the consumption was 1892.6 million tons of oil. Currently, China is facing serious energy poverty [48].

Since 2010, China has changed its position in the world grain market from a net exporter to a net importer. However, increasing grain imports contradicted the Chinese food security policy, which aimed to achieve a grain self-sufficiency of more than 95%. Additionally, Chinese agricultural productivity is lower than the world average. This is an obstacle to the country’s food security target [49].

China is facing huge insecurities of water, energy, and food. So, it is necessary to study how to improve the sustainable development of the WEF system in China. In this study, the authors studied the coupling and coordination degrees of the core WEF nexus of 31 provinces and municipalities in China.

Statistical data from 31 provinces and municipalities were collected from statistical yearbooks as well as the water resources bulletin, which is from official websites of the Statistics Bureau of Provinces and municipalities from 2007 to 2016 [50,51,52,53,54,55,56,57,58,59,60,61,62,63,64,65,66,67,68,69,70,71,72,73,74,75,76,77,78,79,80,81].

## 3. Results and Discussion

The three key parts of this study focused on the following: (1) the development level of the water, energy, and food subsystems; (2) the coupling degree of the core WEF nexus in China, and (3) the coordination degree of the core WEF nexus in China. The results are discussed in detail below. The weights and contributions of indexes are shown in Table 4.

The entropy value method was used to calculate the contribution of each indicator to the development level of each subsystem.

(13) a = 0.313,  b = 0.222,  c = 0.465

Equation (13) indicated that the food subsystem was more important to the development level of the WEF system in this study than other subsystems.

### 3.1. The Development Level of the Core WEF Nexus

**Figure 2:**
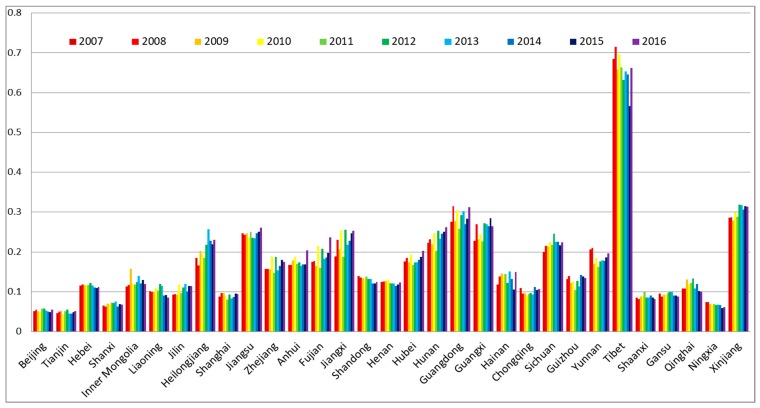
The development levels of the water subsystem of 31 provinces and municipalities in China.

**Figure 3:**
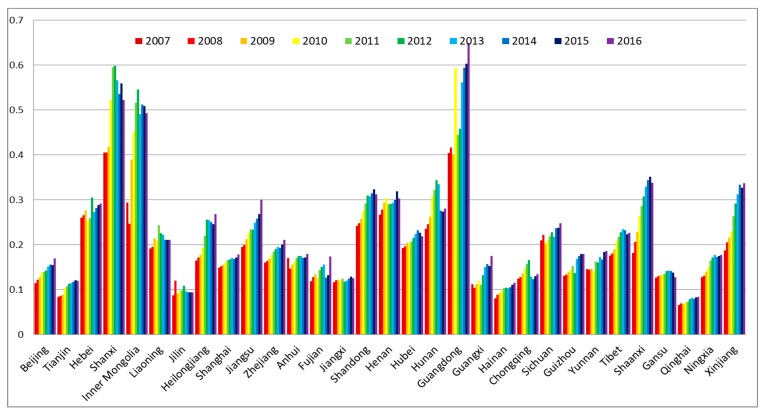
The development levels of the energy subsystem of 31 provinces and municipalities in China.

**Figure 4:**
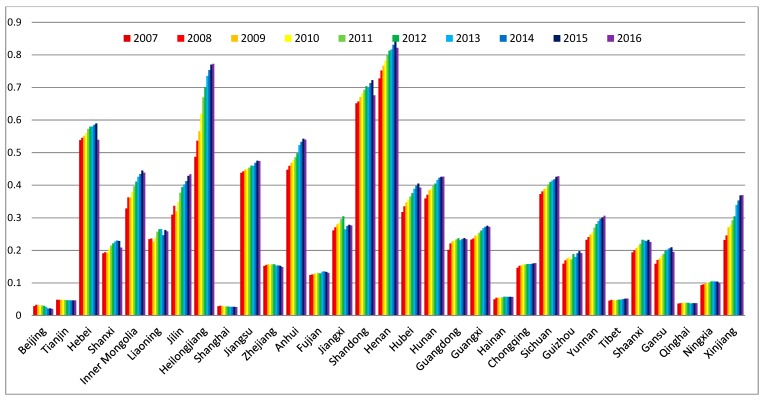
The development levels of the food subsystem of 31 provinces and municipalities in China.

In this study, the development levels of water, energy, food subsystems were calculated first. These results showed the development levels of the three resource subsystems in 31 provinces and municipalities of China in the period from 2007 to 2016 (see Figure 2, Figure 3 and Figure 4).

According to Figure 2, during the selected years from 2007 to 2016, the development level of the water subsystem in each province and municipality barely changed. In a spatial view, the development levels of the water subsystem in Beijing, Tianjin, Shanxi, and Ningxia were low; while in Tibet, Xinjiang, Guangdong, Guangxi, Jiangsu, Hunan, Yunnan, Sichuan, Jiangxi, and Heilongjiang, they were relatively high. We found that the total water supply ($f_{12}$) and the total water capital ($f_{11}$) were the key factors that affected the development level of the water subsystem by comparing the index contribution $Q_{1m}$. In the regions where the development levels of the water subsystem were high, such as Xinjiang, Guangdong, and Guangxi, the total water supply and the total water capital were relatively high. Especially, Tibet was not only endowed with high total water capital but also with high average per capita water availability [82].

During the period from 2007 to 2016, the total water supply and the total water capital of each region were steady, this was the reason why the development level of the water subsystems in each region barely changed.

It can be seen from Figure 3 that the development levels of the energy subsystem in most regions steadily rose from 2007 to 2016. The region with the lowest development level of the energy subsystem was Qinghai Province. Development levels of energy subsystems in Shanxi, Inner Mongolia, and Guangdong were notably higher than the other regions. Total energy consumption in primary industry ($f_{23}$) and total energy production ($f_{21}$) were the key factors that affected the development level of the energy subsystem, which was depicted by comparing the index contribution $Q_{2m}$. During the period from 2007 to 2014, because of the increased demand for energy in industrial and domestic sectors, total energy production increased. However, it decreased from 2015 to 2016 because the raw coal output, which plays a major role in total national energy production, greatly decreased while the use of renewable energy sources, such as wind turbines and solar panels, increased [47]. Though the energy structure changed, total energy production showed a growing trend in the selected years, which was the main reason for the increasing development level of the energy subsystem. At the same time, economic growth was one of the reasons for the increasing development level of the energy subsystem [83]. In addition, the two major energy production regions of Shanxi and Inner Mongolia were also major provinces of energy consumption, which made them the regions with a high development level of the energy subsystem. Guangdong Province, with the largest domestic and commercial energy use, was China’s largest energy consumer [47].

According to Figure 4, there was a large gap between the low and high regions. The development levels of the food subsystem in Beijing, Tianjin, Shanghai, Hainan, Tibet, and Qinghai were the lowest, while the development levels in Hebei, Heilongjiang, Shandong, and Henan were high. The regions with a low development level of the food subsystem kept a steady level from 2007 to 2016. However, the regions with a high development level of the food subsystem showed an increasing trend in this period. The irrigable area of arable land ($f_{36}$) and the total food output ($f_{32})$ were the key factors that affected the development level of the food subsystem, which was clearly shown by comparing the index contribution $Q_{3m}$.

Most of the time, arable land depends on water conservancy infrastructures for irrigation. Therefore, water conservancy infrastructures are essential for agricultural development [84]. However, in economically developed regions such as Beijing, Shanghai, and Tianjin, agricultural development is ignored. Their total area of land covered by water conservancy infrastructures is less than other regions. In regions such as Tibet and Qinghai, their natural conditions are not suitable for growing food, which makes their development level in the food subsystem low. On the other hand, urbanization in northern China (such as Hebei, Henan, Shandong, and Heilongjiang) is not as fast as in the southern region; therefore, reduction of cultivated land in the northern region is also relatively slow. Moreover, the irrigable area has shown an increasing trend in recent years. These are the reasons that increasing food production is driven in the northern region [85].

**Figure 5:**
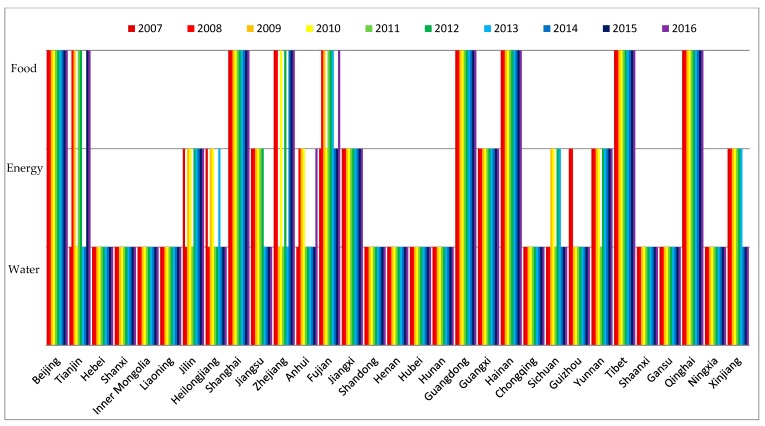
The subsystem with the lowest development level among water, energy, and food subsystems.

Figure 5 shows the subsystem with the lowest development level, among water, energy, and food subsystems, in all selected regions in the period from 2007 to 2016. The development level of the water subsystem was low in most regions. This proved that China is a country facing a serious water crisis. According to statistics, the per capita water resources in China are only 28% of the world average [76]. Water is the main input of energy and food production; therefore, water shortage has become one of the main factors that restricts the development of most regions in China. So, the water subsystem is key in promoting sustainable development of the core WEF nexus.

**Figure 6:**
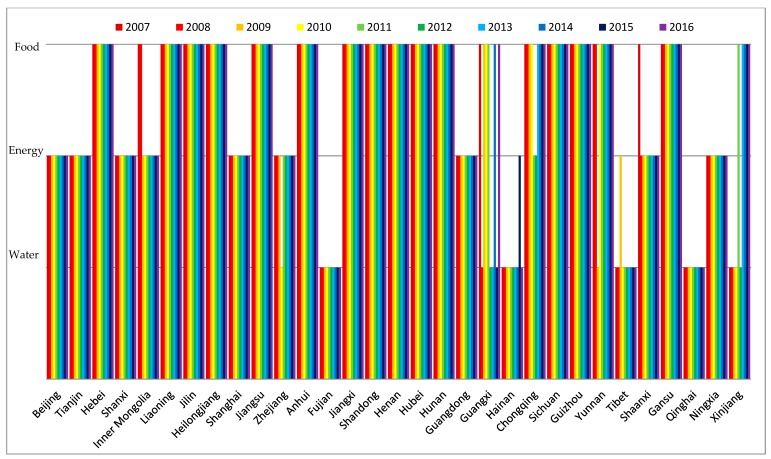
The subsystem with the highest development level among water, energy, and food subsystems.

Figure 6 shows the subsystem with the highest development level, among water, energy, and food subsystems, in all selected regions in the period from 2007 to 2016. As can be seen from Figure 6, in most regions of China, the most developed subsystem was the food subsystem. This proved that China is a largely agricultural country. Though, China also imports large amounts of grain each year, and the ratio of imports has been growing sharply since 2009. At present, China has become the world’s largest food importer [49]. Because of resource constraints such as arable land, water resources, and climate, China’s food security is not guaranteed [85]. Promoting development of the food subsystem is a great challenge in China [86].

### 3.2. The Coupling Degree of the Core WEF Nexus

The coupling levels of the core WEF nexus in the 31 regions of China from 2007 to 2016 is shown in Table 5.

**Figure 7:**
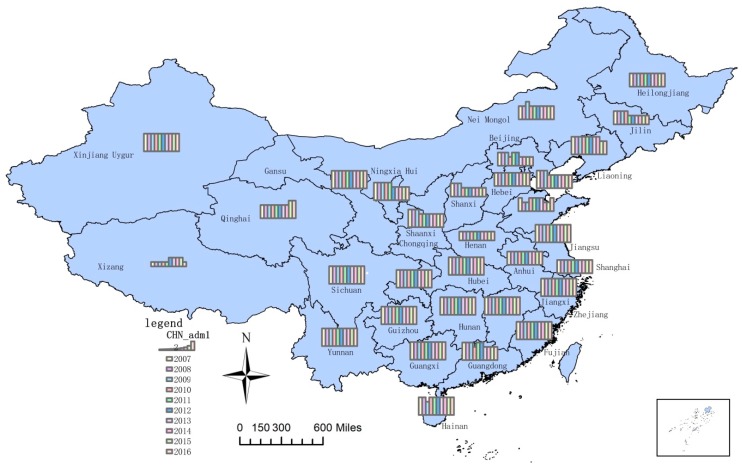
The coupling degree types of the core WEF nexus of 31 regions in China.

According to the classification principle (Table 1), the coupling degree was divided into four types, and the results are shown in Figure 7.

Table 5 shows the coupling degrees of the core WEF nexus in 31 regions of China. Figure 7 presents the coupling degree types (according to Table 3) of the core WEF nexus in all selected regions in China. The higher the histograms are, the higher the coupling degrees are. As can be seen from Table 5 and Figure 7, the coupling degrees of the core WEF nexus in most regions in China were in the high or very high level and kept a steady trend. From Equation (10) we saw that the closer the development levels of the three subsystems were, the higher the coupling degree was. Tibet was the only region with a low or very low coupling level, while its coupling degree rose since 2012. This was because the development levels of the energy and food subsystems in Tibet increased, which narrowed the gap between them and the water subsystem. [87] On the contrary, the increase in the development level of the energy subsystem in Shaanxi made the gap between the water and energy subsystems widen.

In terms of timing, the coupling degrees in most regions of China were steady from 2007 to 2016. There were a few regions, such as Tibet, Shaanxi, and Jilin, in which the coupling degree changed. Spatially, the coupling degrees of most regions were high, except for Tibet and Henan. Consequently, there was a strong coupling reaction among the three subsystems. The water, energy, and food subsystems were interdependent; hence, it was significantly important to study the WEF nexus.

### 3.3. The Coordination Degree of the Core WEF Nexus

The coordination levels of the core WEF nexus in China from 2007 to 2016 is shown in Table 6.

**Figure 8:**
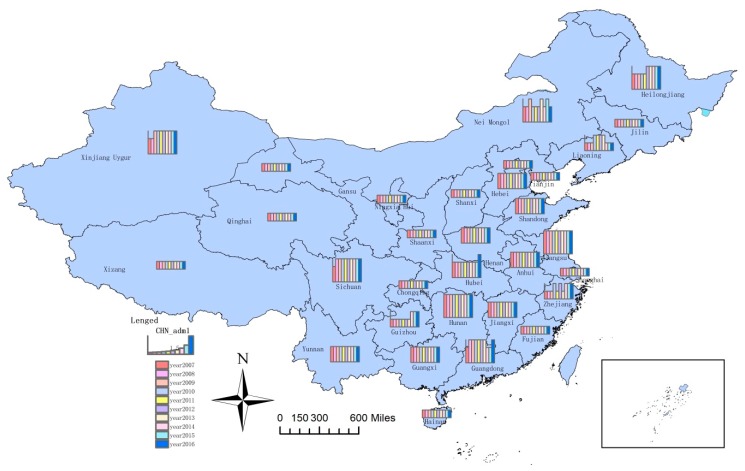
The coordination degree types of the core WEF nexus of 31 regions in China.

According to the classification principle (Table 2), the coordination degree was divided into four types, and the results are shown in Figure 8.

Table 6 shows the coordination degrees of the core WEF nexus in 31 regions of China. Figure 8 presents the coordination degree types (according to Table 4) of the core WEF nexus in all selected regions in China. The higher the histograms are, the higher the coordination degrees are. As can be seen from Table 6 and Figure 8, from 2007 to 2016, the coordination degrees of the core WEF nexus in most regions were either low or medium and were steady. Spatially, coordination degrees were very low for nearly half of the regions, and the regions with high coordination degrees were few. According to Equation (11), the coordination degree depended on the coupling degree and the integrated level of the core WEF nexus, and they were positively correlated. The coupling degree could be improved by increasing the lowest development level to narrow the gap between the three subsystems. Although improving the development levels of the three resources were important to improve the integrated level of the core WEF nexus, the subsystem with the lowest development level should be given more attention.

In general, the coordination degree of the core WEF nexus in most regions of China remained basically unchanged from 2007 to 2016. Meanwhile, the overall coordination degree was low, resulting in the uneven development of water, energy, and food systems. It seriously hindered sustainable development of the WEF system.

As a result, the development level varied greatly throughout the country. The development level of the water subsystem was greatly affected by the average per capita water availability; the development level of the energy subsystem was highly affected by the total energy production; and the development level of the food subsystem was impacted by the degree of agricultural mechanization and the availability of arable land.

From 2007 to 2016, a relatively high coupling degree and a low coordination degree of the core WEF nexus was seen in China. Many provinces and municipalities were still in a low coordination degree, which seriously impeded synergetic development of the WEF nexus. Moreover, during the period from 2007 to 2016, development levels of the three resources changed little, which led to steady coupling and coordination degrees of the core WEF nexus. It is necessary to take some measures to improve the sustainability of the WEF system.

In a nutshell, since the three resources are interdependent and closely related, integrated management of water, energy, and food systems is of great significance to social development, resource sustainability, and ecological protection. For this reason, in order to ensure sustainable development, proper attention should be given to designing synergetic development plans for WEF systems.

## 4. Conclusions

Sustainable development depends on the coordinated development of the WEF system. Complex linkages between the water, energy, and food subsystems pose a great challenge in coordinating their development [2]. At present, the coordination degrees of WEF systems in 31 provinces and municipalities of China are low. Results showed that the water subsystem was key in improving the coupling and coordination degrees of the WEF system. We also found that total water supply, total water capital, total energy consumption in primary industry, energy production, the irrigable area of arable land, and food total output were key factors that affected development levels of the three subsystems.

Based on the analysis above, the following suggestions are put forward to promote synergetic development of the core WEF nexus in China. (1) Take appropriate measures to gradually turn to industries that are high-yield, low-energy, and environmentally friendly [35]. (2) Promote inter-regional sharing of resources, and establish reasonable distribution and utilization mechanisms throughout the country, so that the overall social benefits can be maximized through inter-regional resource collaboration [34]. (3) Improve the utilization efficiency of water in food production by optimizing allocation of water resources and building agricultural infrastructure and water conservancy facilities. (4) Adopt dynamic resource management policies, and take into account factors such as population growth, climate change, and urbanization rate. (5) Synergistically manage water, energy, and food resources, and improve balanced development of the three subsystems. (6) Establish inter-departmental dialogue and consultation mechanisms to help decision-makers formulate feasible and comprehensive policies.

This study identified key factors that affect development of the water, energy, and food subsystem. The water subsystem is the key to improve the coordination degree of the core WEF nexus of 32 regions of China. Hence, the authors believe that this work furthers the efforts to find an approach that promotes the sustainable development of the WEF system in China.

It should be noted that this study has examined only the core WEF nexus and not the peripheral nexus. Hence, social, economic, and environmental subsystems were not taken into account, and care should be taken when interpreting the results. Further studies are necessary to fully understand the coupling and coordination degrees of the WEF nexus.

## Figures and Tables

**Table 1:** Evaluation Index System of Coupling and Coordination Degree of the Core WEF Nexus.

$\mathrm{The}\;\mathrm{First}\;\mathrm{Indicators}\;(f_{i})$	$\mathrm{The}\;\mathrm{Secondary}\;\mathrm{Indicators}\;(f_{im})$	Unit	Attribute of Index
Water Subsystem (f_1_)	Total water resources ($f_{11}$)	10^8^ m^3^	positive
	Total water supply ($f_{12}$)	10^8^ m^3^	positive
	Amount of precipitation ($f_{13}$)	mm	positive
	Average per capita water resources (f_14_)	m^3^	positive
	Agricultural water consumption ($f_{15}$)	10^8^ m^3^	positive
	Irrigation of farmland uses water per mu ($f_{16}$)	m^3^	positive
	Industrial water consumption ($f_{17}$)	10^8^ m^3^	negative
	Residential water consumption ($f_{18}$)	10^8^ m^3^	negative
Energy Subsystem (f_2_)	Total energy production ($f_{21}$)	10^4^ tce	positive
	Total energy consumption ($f_{22}$)	10^4^ tce	positive
	Total energy consumption in primary industry ($f_{23}$)	10^4^ tce	positive
	Total energy consumption in water production and supply industries ($f_{24}$)	10^4^ tce	positive
	Total industrial energy consumption ($f_{25}$)	10^4^ tce	negative
Food Subsystem (f_3_)	Food acreage ($f_{31}$)	10^3^ hm^2^	positive
	Food total output ($f_{32}$)	10 kiloton	positive
	Food output per unit area ($f_{33}$)	Kg/hectare	positive
	Per capita output of food ($f_{34}$)	Kg	positive
	Total power of agricultural machinery ($f_{35}$)	GW	positive
	Irrigable area of arable land ($f_{36}$)	10^3^ hm^2^	positive
	Amount of fertilizer applied to agriculture ($f_{37}$)	10^4^ ton	positive

**Table 2:** Four Types of Coupling Degrees.

$\mathrm{The}\;\mathrm{Type}\;\mathrm{of}\;\mathrm{Coupling}\;\mathrm{Degree}\;C_{rn}$	Coupling Stage	Coupling Range
1	Very Low	(0, 0.3]
2	Low	(0.3, 0.5]
3	High	(0.5, 0.8]
4	Very High	(0.8, 1]

**Table 3:** Four Types of Coordination Degree.

The Type of Coordination Level	Coordination Stage	Coordination Range
1	Low	(0, 0.4]
2	middle	(0.4, 0.5]
3	High	(0.5, 0.8]
4	Extreme high	(0.8, 1)

**Table 4:** Weights of Indexes.

$\mathrm{The}\;\mathrm{Primary}\;\mathrm{Indicators}\;(f_{i})$	$\mathrm{The}\;\mathrm{Secondary}\;\mathrm{Indicators}\;(f_{im})$	$\mathrm{Weight}\;(w_{m})$	$\mathrm{Contribution}\;(Q_{im})$
**Water Subsystem (** $f_{1}$ **)**	Total water capital ($f_{11})$	0.159	0.188
	Total water supply ($f_{12})$	0.117	0.215
	Amount of precipitation ($f_{13})$	0.067	0.147
	Average per capita water availability ($f_{14})$	0.498	0.153
	Agricultural water consumption ($f_{15})$	0.122	0.155
	Irrigation of farmland uses water per mu ($f_{16})$	0.021	0.061
	Industrial water consumption ($f_{17})$	0.007	0.037
	Residential water consumption ($f_{18})$	0.009	0.044
**Energy Subsystem (** $f_{2}$ **)**	Total energy production ($f_{21})$	0.362	0.252
	Total energy consumption ($f_{22})$	0.147	0.222
	Total energy consumption in primary industry ($f_{23})$	0.184	0.257
	Total energy consumption in water production and supply industries ($f_{24})$	0.269	0.144
	Total industrial energy consumption ($f_{25})$	0.038	0.124
**Food Subsystem (** $f_{3}$ **)**	Food acreage ($f_{31})$	0.167	0.178
	Food total output ($f_{32})$	0.181	0.187
	Food output per unit area ($f_{33})$	0.010	0.025
	Per capita output of food ($f_{34})$	0.117	0.104
	The total power of agricultural machinery ($f_{35})$	0.191	0.158
	Irrigable area of arable land ($f_{36})$	0.174	0.202
	Amount of fertilizer applied to agriculture ($f_{37})$	0.160	0.146

**Table 5:** The coupling levels of the core WEF nexus of 31 regions in China.

	Year	2007	2008	2009	2010	2011	2012	2013	2014	2015	2016
Regions											
Beijing		0.602	0.619	0.563	0.490	0.534	0.518	0.423	0.354	0.353	0.331
Tianjin		0.877	0.876	0.881	0.756	0.773	0.765	0.703	0.688	0.688	0.711
Hebei		0.582	0.585	0.587	0.550	0.547	0.598	0.555	0.549	0.549	0.603
Shanxi		0.506	0.505	0.509	0.413	0.373	0.374	0.420	0.421	0.413	0.415
Inner Mongolia		0.793	0.762	0.835	0.723	0.686	0.684	0.744	0.697	0.716	0.702
Liaoning		0.860	0.849	0.857	0.873	0.830	0.868	0.856	0.810	0.800	0.783
Jilin		0.508	0.558	0.507	0.545	0.440	0.483	0.462	0.387	0.407	0.399
Heilongjiang		0.639	0.558	0.595	0.556	0.532	0.600	0.619	0.563	0.533	0.572
Shanghai		0.599	0.624	0.607	0.570	0.525	0.556	0.510	0.528	0.548	0.518
Jiangsu		0.822	0.820	0.833	0.836	0.854	0.833	0.847	0.858	0.864	0.896
Zhejiang		0.999	0.999	0.998	0.991	0.986	0.989	0.979	0.986	0.981	0.969
Anhui		0.667	0.608	0.633	0.650	0.622	0.622	0.578	0.565	0.555	0.630
Fujian		0.953	0.959	0.982	0.899	0.990	0.940	0.977	0.954	0.943	0.916
Jiangxi		0.859	0.868	0.857	0.849	0.826	0.828	0.871	0.870	0.875	0.867
Shandong		0.501	0.497	0.495	0.503	0.522	0.519	0.521	0.498	0.500	0.531
Henan		0.442	0.441	0.449	0.455	0.415	0.404	0.404	0.392	0.406	0.416
Hubei		0.890	0.882	0.860	0.875	0.829	0.836	0.828	0.833	0.830	0.856
Hunan		0.925	0.928	0.914	0.950	0.900	0.949	0.924	0.912	0.913	0.924
Guangdong		0.883	0.909	0.916	0.763	0.870	0.883	0.790	0.735	0.743	0.725
Guangxi		0.877	0.827	0.867	0.879	0.860	0.882	0.913	0.925	0.906	0.949
Hainan		0.840	0.812	0.797	0.814	0.820	0.884	0.812	0.859	0.899	0.823
Chongqing		0.979	0.947	0.948	0.928	0.931	0.927	0.934	0.966	0.955	0.960
Sichuan		0.867	0.887	0.858	0.872	0.863	0.888	0.856	0.870	0.854	0.868
Guizhou		0.988	0.983	0.965	0.967	0.942	0.953	0.948	0.978	0.969	0.969
Yunnan		0.949	0.940	0.922	0.915	0.905	0.902	0.907	0.891	0.913	0.918
Tibet		0.199	0.194	0.235	0.238	0.274	0.309	0.304	0.309	0.357	0.295
Shaanxi		0.857	0.824	0.822	0.790	0.795	0.733	0.709	0.704	0.683	0.685
Gansu		0.938	0.900	0.909	0.896	0.894	0.887	0.885	0.849	0.841	0.850
Qinghai		0.764	0.780	0.685	0.749	0.742	0.714	0.791	0.757	0.819	0.820
Ningxia		0.923	0.918	0.882	0.852	0.821	0.800	0.790	0.790	0.753	0.752
Xinjiang		0.957	0.973	0.982	0.985	0.997	0.998	0.998	0.995	0.993	0.993

**Table 6:** Coordination levels of the core WEF nexus of 31 regions in China.

	Year	2007	2008	2009	2010	2011	2012	2013	2014	2015	2016
Regions											
Beijing		0.182	0.192	0.183	0.172	0.184	0.181	0.162	0.146	0.145	0.145
Tianjin		0.22	0.224	0.227	0.210	0.219	0.221	0.208	0.206	0.209	0.214
Hebei		0.447	0.452	0.456	0.440	0.444	0.474	0.451	0.450	0.452	0.460
Shanxi		0.317	0.318	0.323	0.310	0.308	0.311	0.327	0.322	0.323	0.312
Inner Mongolia		0.449	0.445	0.504	0.477	0.480	0.490	0.510	0.496	0.508	0.496
Liaoning		0.397	0.395	0.397	0.409	0.413	0.427	0.422	0.392	0.397	0.389
Jilin		0.313	0.345	0.317	0.347	0.316	0.342	0.337	0.307	0.323	0.321
Heilongjiang		0.453	0.435	0.466	0.468	0.472	0.520	0.545	0.519	0.507	0.530
Shanghai		0.210	0.220	0.218	0.210	0.198	0.209	0.197	0.202	0.210	0.205
Jiangsu		0.516	0.517	0.526	0.527	0.540	0.532	0.538	0.550	0.557	0.575
Zhejiang		0.394	0.397	0.399	0.412	0.397	0.415	0.399	0.405	0.411	0.407
Anhui		0.446	0.426	0.442	0.453	0.444	0.449	0.439	0.437	0.436	0.473
Fujian		0.364	0.370	0.372	0.373	0.376	0.386	0.388	0.378	0.381	0.399
Jiangxi		0.421	0.442	0.436	0.450	0.430	0.453	0.436	0.445	0.454	0.452
Shandong		0.448	0.447	0.451	0.460	0.474	0.476	0.476	0.467	0.472	0.474
Henan		0.439	0.446	0.455	0.464	0.443	0.440	0.442	0.438	0.452	0.451
Hubei		0.468	0.477	0.474	0.487	0.472	0.481	0.486	0.494	0.497	0.502
Hunan		0.517	0.527	0.529	0.556	0.537	0.571	0.561	0.553	0.555	0.562
Guangdong		0.488	0.517	0.507	0.505	0.501	0.517	0.508	0.489	0.497	0.504
Guangxi		0.424	0.424	0.428	0.438	0.429	0.456	0.470	0.475	0.477	0.486
Hainan		0.256	0.267	0.270	0.273	0.278	0.279	0.280	0.280	0.275	0.285
Chongqing		0.357	0.350	0.353	0.352	0.358	0.361	0.350	0.362	0.360	0.364
Sichuan		0.495	0.510	0.501	0.512	0.512	0.531	0.517	0.526	0.522	0.530
Guizhou		0.378	0.387	0.381	0.387	0.372	0.388	0.385	0.410	0.412	0.407
Yunnan		0.441	0.444	0.431	0.436	0.439	0.447	0.456	0.454	0.467	0.474
Tibet		0.234	0.236	0.252	0.261	0.276	0.289	0.292	0.293	0.299	0.288
Shaanxi		0.367	0.368	0.379	0.384	0.396	0.386	0.383	0.385	0.381	0.375
Gansu		0.351	0.349	0.356	0.358	0.364	0.370	0.373	0.364	0.363	0.353
Qinghai		0.224	0.228	0.225	0.231	0.232	0.235	0.234	0.234	0.235	0.235
Ningxia		0.296	0.298	0.294	0.294	0.294	0.294	0.294	0.292	0.282	0.281
Xinjiang		0.478	0.493	0.506	0.520	0.533	0.553	0.571	0.577	0.583	0.585
